# Deciphering the Pleiotropic Role of *ARID1a* and *RIF1* in Endometrioid Ovarian Cancer

**DOI:** 10.3390/cells15111036

**Published:** 2026-06-04

**Authors:** Pawel Kordowitzki, Renata Voltolini Velho, Sandra Bock, Jalid Sehouli, Sylvia Mechsner

**Affiliations:** 1 Department of Basic and Preclinical Sciences, Nicolaus Copernicus University, 87-100 Torun, Poland; 2 Institute of Advanced Studies, Nicolaus Copernicus University, 87-100 Torun, Poland; 3 Department of Gynecology with Center for Oncological Surgery, Campus Virchow Klinikum, Charité-Universitätsmedizin Berlin, 13353 Berlin, Germany

**Keywords:** *ARID1a*, *BRCA1*, *RIF1*, *LAMB3*, endometrioid ovarian cancer, endometriosis

## Abstract

**Highlights:**

**What are the main findings?**

- Endometrioid ovarian cancer exhibited significantly reduced BAF250a/ARID1a protein expression and increased RIF1 expression.
- Specific oncogenic mutations, including *BRCA1* and *LAMB3*, were significantly associated with altered *ARID1a* or *RIF1* expression.

**What are the implications of the main findings?**

- *RIF1* expression demonstrated significant predictive value for platinum- and taxane-based treatment response.
- Identification of *KIF17* as a potential positive regulator of *ARID1a* expression provides new avenues for understanding the upstream oncogenic events of endometrioid ovarian cancer

**Abstract:**

Background: Given the challenges in early detection and diagnosis, understanding the molecular underpinnings of endometrioid ovarian cancer (EOC) is crucial for improving patient outcomes. This multi-level study provides a new perspective on EOC, focusing on the expression of *ARID1a* (BAF250a) and *RIF1*. Methods: This study evaluates patient cohorts with EOC through semi-quantitative immunohistochemical staining of BAF250a (protein encoded by *ARID1a*) and RIF1 proteins alongside mutations that influence the gene expression of *ARID1a* and *RIF1*. Besides survival analyses, platinum- and taxane-based treatment responsiveness with regard to *ARID1a* and *RIF1* expression has been analyzed using an online available database. Results: Histological and immunohistochemical analysis of clinical samples revealed a significant reciprocal alteration in protein expression, characterized by a marked reduction in the tumor suppressor BAF250a (*p* < 0.0001) and a concomitant elevation of RIF1 (*p* < 0.0001) in EOC compared to controls. Tumors harboring mutations in *BRCA1* exhibited significantly (*p* = 2.82 × 10^−4^) lower *ARID1a* expression levels compared with corresponding wild-type tumors, whereas *LAMB3*-mutant tumors showed a significant (*p* = 5.16 × 10^−3^) upregulation of *RIF1* mRNA expression. Conclusions: In conclusion, our study offers a new perspective, emphasizing that EOC is a distinct clinical and molecular entity. We demonstrated the expression patterns of ARID1a/BAF250a and RIF1 in EOC, establishing their potential relevance in the context of tumor biology and malignant transformation.

## 1. Introduction

Endometrioid ovarian cancer (EOC) constitutes approximately 20–40% of epithelial ovarian cancers and is one subtype of endometriosis-associated ovarian cancers [1,2]. The global incidence rates demonstrate variability, with EOC prevalence ranging from 7% to 13% in surgical series [3]. The EOC subtype is notable for its resemblance to endometrial carcinoma of the uterus and a frequent association with endometriosis [4]. This association suggests a potential adenoma–carcinoma progression pathway, in which benign endometrioid lesions may progress to well-differentiated carcinomas [5]. Notably, although endometriosis is generally considered a benign gynecological disorder, its potential for malignant transformation into the endometrioid subtype underscores its clinical importance [6,7,8]. This connection has prompted extensive research into shared molecular pathways and key driver genes involved in their pathogenesis [9]. There is no doubt that the pathogenesis of EOC is multifactorial, involving a complex interplay of genetic, molecular, and environmental factors. Key mechanisms include genetic mutations, such as those in *ARID1a*, which are frequently observed in EOC and contribute to the dysregulation of oncogenic pathways [10,11,12]. *ARID1a* is a crucial component of the SWI/SNF (Switch/Sucrose Non-Fermentable) chromatin remodeling complex, which plays a vital role in epigenetic reprogramming and gene expression [13,14]. As a tumor suppressor, *ARID1a* is frequently mutated in ovarian clear cell carcinomas (over 50%) and EOC (around 30%), with these mutations typically leading to a loss of BAF250a protein expression [10,15,16,17,18]. Another gene of interest in the pathogenesis of EOC might be *RIF1* (Rap1-interacting factor 1), as its expression is significantly upregulated in epithelial ovarian cancer and associated with platinum resistance and poor prognosis [18,19,20]. The RIF1 protein actively promotes the growth and progression of epithelial ovarian cancer cells [20]. Its involvement in DNA repair pathways and the activation of telomerase reverse transcriptase (hTERT) highlights RIF1’s importance in telomere biology, maintaining genomic stability and cancer stem cell characteristics, making it a promising biomarker and potential therapeutic target [21,22,23]. Current serum tumor markers, such as CA125, lack the necessary specificity and sensitivity for early EOC detection, as their levels can be influenced by various benign conditions, including endometriosis itself [24,25]. Understanding the molecular interplay between aging and EOC development is crucial for identifying novel diagnostic markers and therapeutic strategies to improve outcomes in this challenging disease [25].

We hypothesize that a reciprocal regulatory relationship exists between chromatin remodeling dysfunction and telomere repair signaling specifically in the endometriosis-associated subtype, driven by chronic inflammatory stress and replication-associated DNA damage. In this context, loss or dysregulation of a chromatin remodeler such as *ARID1A* may induce compensatory upregulation of telomere-associated DNA repair factors, including RIF1, in order to maintain genome stability under persistent oxidative and replicative stress. Conversely, sustained activation of telomere repair pathways may further reshape chromatin accessibility and epigenetic organization, thereby promoting survival of genomically unstable endometriotic epithelial cells. Therefore, the aim of this retrospective study was to elucidate how the expression of *ARID1a* and *RIF1* in EOC is altered and to clarify whether these genes could be key drivers of the malignant transformation of endometriosis into EOC.

## 2. Materials and Methods

### 2.1. Ethical Approval

The study was approved by the ethics commissions of Charité—Universitätsmedizin Berlin (EA2/266/22 and EA1/150/24). Formalin-fixed paraffin-embedded (FFPE) tissues from patients were acquired with informed consent following the local institutional review and the Declaration of Helsinki.

### 2.2. Study Design

This retrospective study evaluated patients with histologically confirmed endometrioid ovarian cancer (FIGO stages IIIC-IVA, grade 2–3, no neoadjuvant chemotherapy before the surgery at the time of primary treatment, age range 35–65 years) and endometriosis (endometrioma, ENZIAN P1-P3) patients (age range 19–60 years). The study was conducted at Charité—Universitätsmedizin Berlin, a German tertiary academic medical center and certified European Society of Gynecological Oncology (ESGO) Center of Excellence.

Inclusion Criteria: Eligible patients were those who received primary treatment for an EOC tumor and for endometriosis at the Department of Gynecology of the Charité—Universitätsmedizin Berlin between 1 January 2014 and 31 July 2025.

Exclusion criteria: Criteria for exclusion from the study cohort included primary surgical intervention performed at an external institution, the presence of ovarian metastases derived from non-ovarian primary sites, and the existence of insufficient clinicopathologic or longitudinal follow-up data.

Study participants were ascertained via the institutional cancer registry, with pertinent clinical, histopathological, and therapeutic parameters systematically retrieved from electronic medical records and the institutional pathology database. Moreover, for the survival analysis, treatment response probability, and mutation status evaluation, the online Database on CancerHallmarks.com has been used.

### 2.3. Immunohistochemical Staining

Tissue quality assessment for cellularity and morphology, prior to immunohistochemical staining, was assessed based on the evaluation of Hematoxylin and Eosin (H&E) stained slides. An experienced pathologist examined the samples to ensure it contains a high enough percentage of target cells (such as tumor cells) relative to the stroma. Four-micrometer tissue sections were mounted on TOMO slides (*n* = 30) (Matsunami Glass, TOM-1190, Matsunami Glass Ind., Ltd., Kishiwada City, Japan) and subjected to immunohistochemical staining using Benchmark stainers (Roche, Ventana Medical Systems, Tucson, USA). BAF250a protein staining was performed on a Benchmark Discovery Ultra, with antigen retrieval using CC1 standard buffer. Sections were incubated with the primary antibody (Abcam, ab182560, clone EPR13501, 1:1000, Cambridge, UK) for 60 min, followed by a secondary anti-rabbit antibody (Vector, BA-1000-1.5, 1:200, Merck, Darmstadt, Germany) for 30 min. Visualization employed the DISCOVERY DAB Map Kit (Roche, 760-124, Ventana Medical Systems, Tucson, AZ, USA), and nuclei were counterstained with hematoxylin and bluing reagent. RIF1 staining was performed on a Benchmark Ultra, followed by primary antibody incubation (Abcam, ab229632, 1:500) for 32 min. Visualization was achieved using the ultraView Universal DAB Detection Kit (Roche, 760-500, Ventana Medical Systems, Tucson, AZ, USA), and the same nuclear counterstaining protocol was applied. All stained slides were digitized using a Leica Aperio GT 450 DX scanner (Leica Microsystems GmbH, Wetzlar, Germany) for analysis. An experienced pathologist screened the slides for fixation artifacts and excluded those if the sample suffered from over-fixation or under-fixation. The percentage of microscopically positive cells and staining intensity was scored by semi-quantitative interpretation. Five fields of view were observed on each section. In total, 15 EOC samples and 13 endometriosis samples (endometriosis genitalis externa, EZIAN 3) were analyzed by one examiner in a blinded manner. IHC evaluation quantified protein expression by combining the percentage of positive cells with the staining intensity. Staining intensity (degree of staining) refers to the grade of darkness or the concentration of the chromogen (DAB). The intensity of positive staining is also graded on a scale of 0 to 3, where colorless is 0, pale yellow is 1, tan is 2, and brown is 3. The percentage of positive cells evaluates the proportion of target cells that exhibit any visible staining. The percentage of positive-stained cells is graded on a scale of 0 to 4, where <5% is 0, 5–25% is 1, 26–50% is 2, 51–75% is 3, and 76–100% is 4. The positive grade is obtained by multiplying the two scores and relies on a standardized grading system that is widely used and well-established.

### 2.4. Kaplan–Meier Survival Probability Analysis

The gene-specific Kaplan–Meier analysis was performed with the help of the recently published online plotter tool and the respective database and restricted to endometrioid ovarian cancers [26]. Briefly, survival analysis was performed using the lifelines (v.0.27.4) package in Python v.3.10. Inclusion in the analysis was restricted to genes associated with at least 5 relapse events in at least one of the cohorts. Survival outcomes were characterized using Cox proportional hazards models and Log-Rank tests to evaluate prognostic significance.

### 2.5. Identification of Mutations Influencing the Expression of ARID1a and RIF1

The muTarget cancer biomarker platform and its integrated database were utilized to evaluate the impact of somatic mutations on the mRNA expression profiles of *ARID1a* and *RIF1*. These analyses were conducted in accordance with the methodological framework previously established and described by Nagy and Gyorffy [27]. In brief, algorithm development and data processing were implemented utilizing R software (v3.5.2). Somatic mutation datasets, identified through the Mutect2 pipeline, and corresponding transcriptomic profiles were retrieved from the Cancer Genome Atlas repository. Data aggregation and summarization were conducted via the MAFtools package within the R Bioconductor environment. To facilitate the analysis of cancer-specific somatic alterations, Mutation Annotation Format files were employed, enabling the systematic exclusion of low-confidence signals and putative germline variants. Mutation filtering was informed by Mutect2 classifications and further refined by stringent criteria, requiring a minimum sequencing depth of 20× and the presence of the identified alteration in at least five reads. Due to the fact that muTarget screens many mutations, we herein focused on the four mutations that most significantly altered the expression of *ARID1a* and *RIF1*.

### 2.6. Platinum- and Taxane-Based Treatment Response with Regard to ARID1a and RIF1 Expression

To evaluate the predictive potential of *ARID1a* and *RIF1* mRNA expression for trastuzumab treatment, the ROC plotter tool and the corresponding database were used, as previously reported [28]. Briefly, study participants were stratified into binary cohorts of treatment responders and non-responders, predicated upon clinical and histopathological response criteria. For individuals undergoing neoadjuvant chemotherapy, a binary classification system was implemented as a substitute for standard four-tier categorization; “responders” were defined by a total absence of histological tumor evidence, while “non-responders” were those with identifiable residual malignant tissue.

Patients in the adjuvant therapy group were stratified based on survival and recurrence status at a five-year follow-up interval. This analysis involved the comparison of gene expression profiles between patients experiencing relapse within five years and those surviving beyond this five-year threshold, with individuals censored before this milestone excluded from the cohort. Statistical evaluations were executed using Mann–Whitney tests or Receiver Operating Characteristic analyses within the R environment, utilizing Bioconductor libraries.

### 2.7. Statistical Analysis

Statistical analyses were conducted using R (v3.5.2) and the lifelines (v.0.27.4) package within a Python (v.3.10) environment. Inter-group comparisons were evaluated via the Mann–Whitney U test or Receiver Operating Characteristic analysis. For the ROC assessments, the optimal diagnostic cut-off threshold was determined by the maximization of the Youden Index. Statistical significance was defined as a *p*-value < 0.05 or a False Discovery Rate < 0.05, as appropriate.

## 3. Results

### 3.1. Histological Appearance and Protein Expression

**Figure 1:**
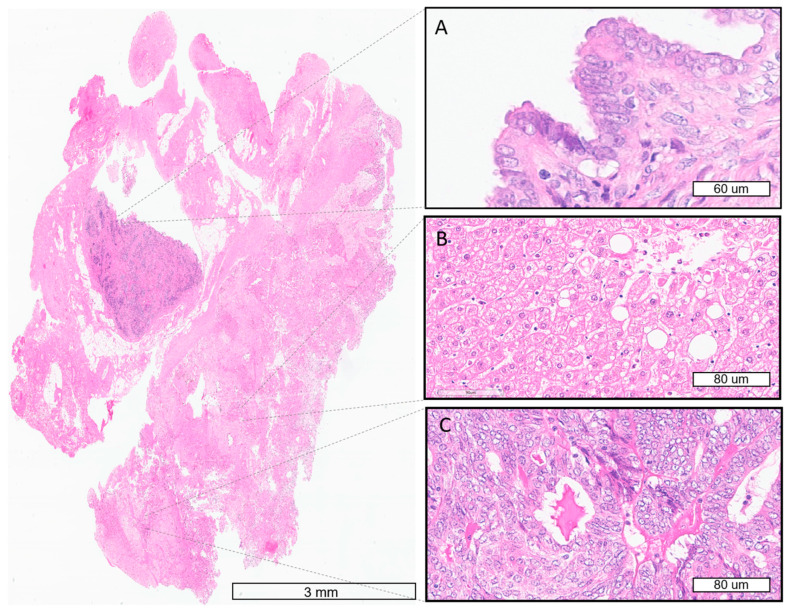
**Representative microscopic picture of endometrioid ovarian cancer**. On the **left**, a hematoxylin and eosin (H&E)-stained resection specimen is shown in which an Endometriosis-Associated Ovarian Cancer has arisen from an endometriotic lesion. The magnified pictures on the **right** show contagious atypical ovarian endometriosis (**Panel A**), endometroid ovarian cancer (**Panel B**), and malignant endometrial glands (**Panel C**).

**Figure 2:**
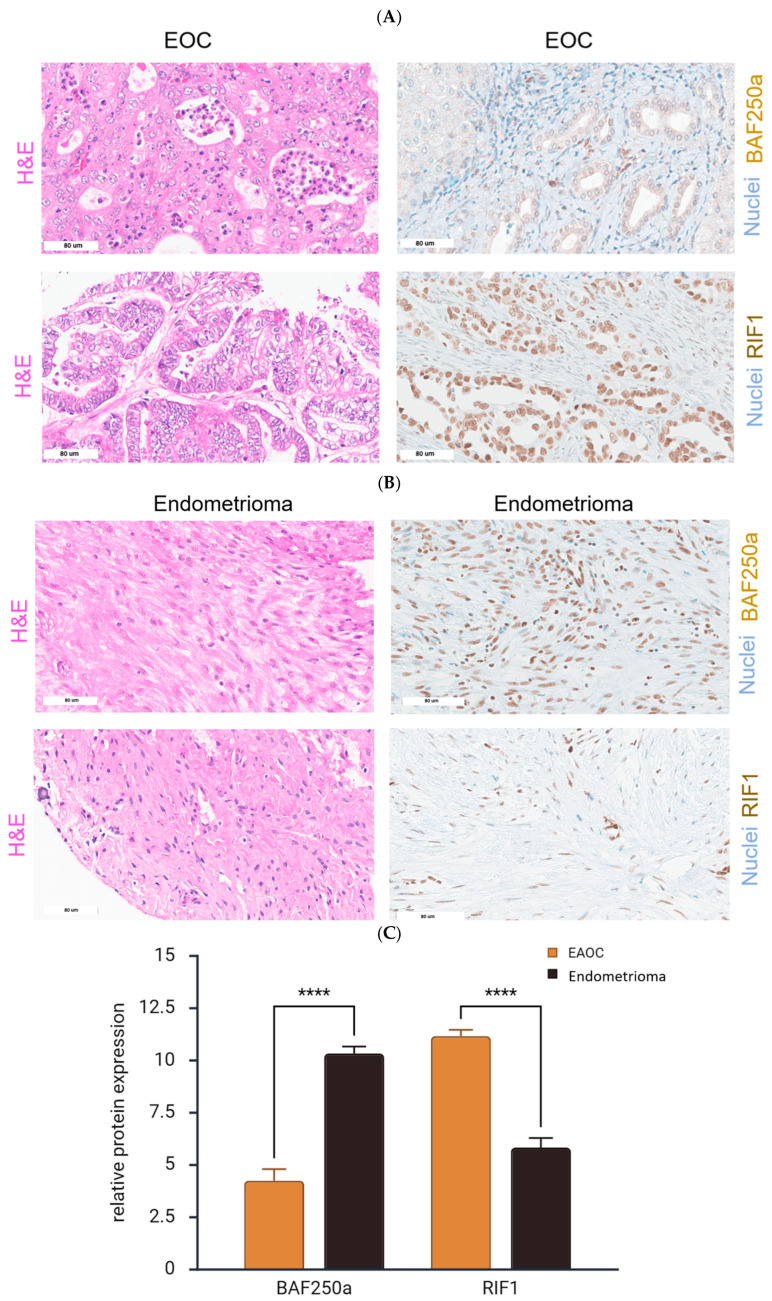
**Protein expression of BAF250a and RIF1.** Panel (**A**) shows representative microscopic images of H&E-stained and immunohistochemical (IHC) protein staining in EOC samples, with positive (brownish stain) BAF250a and RIF1 protein expression. Panel (**B**) shows representative microscopic images of H&E-stained and representative IHC protein staining in Endometrioma samples, with positive (brownish stain) BAF250a and RIF1 protein expression. Panel (**C**) shows the quantitative analyses of protein expression for BAF250a and RIF1. The white scale bar in the lower left corner indicates 80 μm. The *p*-value of < 0.0001 is indicated by ****.

To begin, we analyzed hematoxylin and eosin (H&E)-stained resection sections histologically to confirm the malignant glandular architecture characteristic of EOC (Figure 1). Notably, all ovarian cancers are routinely classified by our Pathology Department based on the gene expression of WT1, PAX8, TP53, ER, PR, and p16. In the next step, immunohistochemical staining of EOC and endometrioma tissue samples was performed to analyze protein expression of BAF250a and RIF1. As shown in Figure 2, a nuclear localization, evident in both the nucleoplasm and the nuclear membrane, was observed for both proteins. Quantitative analysis of stained nuclei revealed a significant reduction (*p* < 0.0001) in BAF250a expression in EOC samples compared with endometriosis samples, which served as controls. In contrast, RIF1 showed a significantly higher protein expression in EAOC compared to control samples (*p* < 0.0001). These findings indicate a reciprocal alteration of chromatin- and DNA repair-associated factors in EOC. In contrast, elevated RIF1 expression suggests that enhanced or deregulated DNA damage response and replication control mechanisms are present.

### 3.2. Survival Probability with Regard to ARID1a and RIF1 Expression

**Figure 3:**
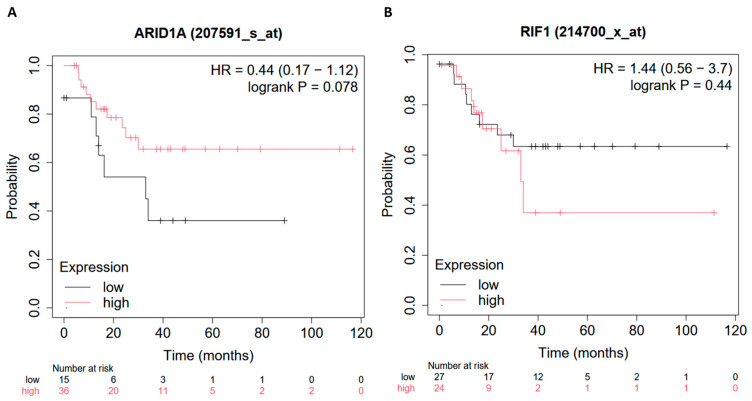
**Kaplan–Meier survival curves with regard to *ARID1a* and *RIF1* expression in stage IV endometrioid ovarian cancer patients.** Panel (**A**) shows the survival probability by *ARID1a* expression status. Kaplan–Meier survival curves stratified by low (black) and high (red) *ARID1a* gene expression. Panel (**B**) shows the survival probability by *RIF1* expression status. Kaplan–Meier survival curves stratified by low (black) and high (red) RIF1 gene expression. Numbers at risk are shown below the plots. Hazard ratios (HRs), 95% confidence intervals, and log-rank *p* values are indicated.

In the survival probability analysis, patients (*n* = 36) with elevated *ARID1a* expression showed no significant difference in overall survival compared with those with low *ARID1a* expression (HR = 0.44, 95% CI: 0.17–1.12; log-rank *p* = 0.078) (Figure 3A). Although the difference does not reach statistical significance, the hazard ratio below 1.0 indicates a relevant effect associated with high *ARID1a* expression. The separation of the survival curves appears early and is maintained over more than 100 months of follow-up. However, the relatively small number of patients at risk at later time points may limit statistical power and contribute to the borderline significance. For the gene *RIF1* (Figure 3B), there is no statistically significant association between its expression levels and survival probability (HR = 1.44, 95% CI: 0.56–3.7; log-rank *p* = 0.44). Here, the hazard ratio above 1 suggests a possible trend toward worse outcomes with high expression, but the wide confidence interval crossing 1.0 and the high *p*-value indicate substantial uncertainty. The survival curves overlap considerably, supporting the conclusion that the expression of *RIF1* is not a robust prognostic factor in this cohort (*n* = 51).

### 3.3. Identification of Mutations Influencing the Expression of ARID1a and RIF1

**Figure 4:**
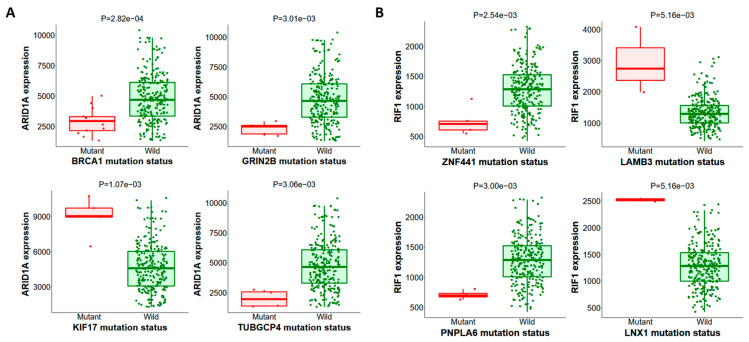
**Differential expression of ARID1a and RIF1 according to the four most significant gene mutations in ovarian cancer.** Boxplots depict the expression levels in the tested cohort (*n* = 272) for mutant versus wild-type tumors. This analysis has been performed in a mixed ovarian cancer cohort and was not restricted to EOC. Panel (**A**) shows *ARID1a* messenger RNA expression stratified by *BRCA1, GRIN2B, KIF17*, and *TUBGCP4* mutation status with the respective *p*-values on the top. Panel (**B**) shows *RIF1* messenger RNA expression stratified by *ZNF441, LAMB3, PNPLA6*, and *LNX1* mutation status, with the respective *p*-values on the top. The Mann–Whitney analysis was used.

The muTarget analysis of ovarian cancer samples (mixed subtypes) revealed significant associations between mutation status and *ARID1A* mRNA expression. Tumors harboring mutations in *BRCA1, GRIN2B,* or *TUBGCP4* exhibited significantly lower *ARID1A* expression levels compared with corresponding wild-type tumors, whereas *KIF17* mutant tumors showed a significant (*p* = 1.07 × 10^−3^) upregulated *ARID1a* expression (Figure 4A). Specifically, *ARID1A* expression was significantly reduced in *BRCA1*-mutant tumors (*p* = 2.82 × 10^−4^), *GRIN2B*-mutant tumors (*p* = 3.01 × 10^−3^), and *TUBGCP4*-mutant tumors (*p* = 3.06 × 10^−3^). In all comparisons, the wild-type groups displayed broader expression distributions and higher median *ARID1A* mRNA expression levels (Figure 4A). The reduced ARID1A expression in tumors harboring *BRCA1*, *GRIN2B*, and *TUBGCP4* mutations suggests a potential convergence of these molecular alterations on pathways regulating chromatin accessibility, DNA repair, or epigenetic stability.

Mutation-dependent alterations were also observed for *RIF1* mRNA expression (Figure 4B). Tumors carrying mutations in *ZNF441 and PNPLA6* demonstrated significantly decreased *RIF1* mRNA expression relative to wild-type tumors, whereas tumors with mutations of the genes *LAMB3* and *LNX1* led to a significant upregulation of *RIF1* mRNA expression. These alterations were highly significant: in *ZNF441*-mutant (*p* = 2.54 × 10^−3^), in *LAMB3*-mutant (*p* = 5.16 × 10^−3^), PNPLA6-mutant (*p* = 3.00 × 10^−3^), and in LNX1-mutant tumors (*p* = 5.16 × 10^−3^). These findings suggest that alterations in *ARID1A* and *RIF1* expression may represent downstream molecular consequences of distinct oncogenic mutational events.

### 3.4. Platinum- and Taxane-Based Treatment Response with Regard to ARID1a and RIF1 Expression

**Figure 5:**
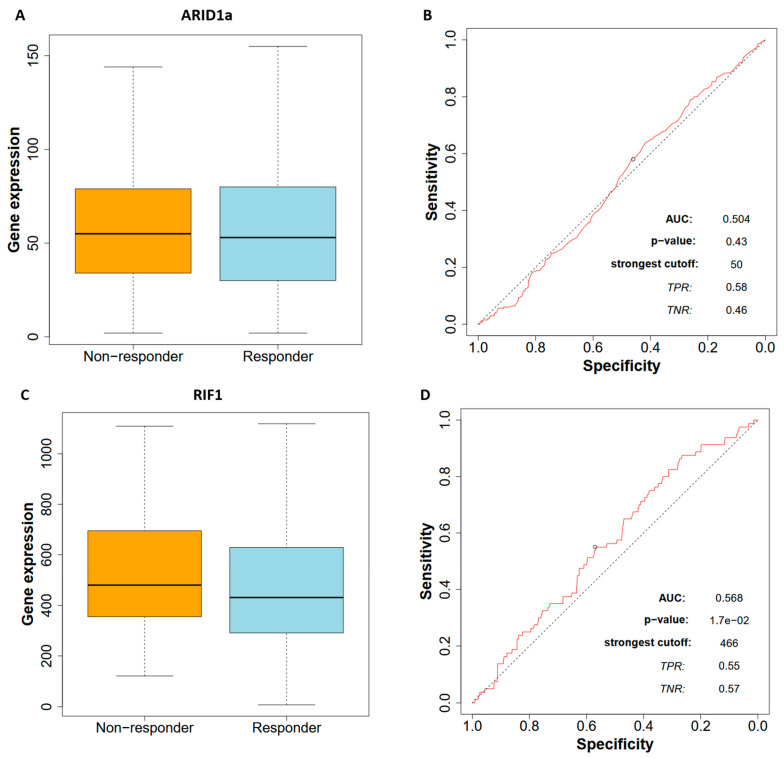
**Treatment responsiveness with regard to *ARID1a* and *RIF1* gene expression.** Relapse-free survival for six months upon platinum and taxane treatment in EOC patients (*n* = 118) is shown. Panel (**A**) shows *ARID1a* expression levels by treatment response status. Panel (**B**) presents a Receiver Operating Characteristic analysis evaluating the utility of ARID1A expression in differentiating treatment responders from non-responders. Utilizing the Youden Index to identify the optimal diagnostic threshold ($cutoff = 50$), the analysis yielded a sensitivity of 0.58 and a specificity of 0.46. Correspondingly, Panel (**C**) depicts RIF1 expression levels according to clinical response. Panel (**D**) illustrates the ROC performance of RIF1 mRNA in predicting therapeutic outcomes; at an optimized cutoff of 466, the marker demonstrated a sensitivity of 0.55 and a specificity of 0.57. The strongest cutoff values were determined using the Youden Index. The two cohorts (responders vs. non-responders) are compared using the Mann–Whitney test and the ROC test in the R statistical environment. Statistical significance was set at *p* < 0.05.

To investigate the association between gene expression and therapeutic outcome, *ARID1a* and *RIF1* expression levels were analyzed EOC patients (*n* = 118) receiving platinum- and taxane-based chemotherapy, stratified according to relapse-free survival at six months following treatment initiation using the online ROC plotter tool. Comparison of *ARID1a* expression between treatment responders and non-responders showed largely overlapping distributions, with no evident separation between the two cohorts (Figure 5A). Median *ARID1a* expression levels were comparable between responders and non-responders, indicating no significant association between *ARID1a* expression and short-term therapeutic response. The ROC analysis evaluating the predictive performance of *ARID1a* expression for treatment response yielded an area under the curve (AUC) of 0.504 with a non-significant *p*-value of 0.43, indicating no discriminatory capacity beyond random classification (Figure 5B). The optimal cutoff value determined by the Youden Index was 50, corresponding to a sensitivity (true-positive rate, TPR) of 0.58 and a specificity (true-negative rate, TNR) of 0.46.

Analysis of *RIF1* expression by treatment response status revealed a trend toward lower expression in treatment responders than in non-responders. Although expression ranges overlapped substantially between groups, responders exhibited a modest reduction in median *RIF1* expression (Figure 5C). The ROC analysis for *RIF1* expression demonstrated statistically significant predictive performance for treatment response, with an AUC of 0.568 and a *p*-value of 1.7 × 10^−2^ (Figure 5D). However, this finding, although significant, should be treated with caution. The optimal cutoff value identified by the Youden Index was 466, yielding a sensitivity of 0.55 and a specificity of 0.57.

## 4. Discussion

This study provides novel insights into the distinct biological characteristics of *RIF1* and *ARID1a* in endometrioid ovarian cancer, underscoring their potential as key drivers in the pathogenesis of EOC. As shown in our study, the opposing patterns of BAF250a downregulation and RIF1 upregulation support the concept that EOC is characterized by coordinated epigenetic dysregulation and altered genome maintenance pathways, which may contribute to tumor development and progression. Immunohistochemical analysis revealed differential protein expression of BAF250a (encoded by *ARID1a*) and RIF1 in EOC tumor samples and respective controls (endometrioma). Notably, *ARID1a* functions as a tumor suppressor. Its recurrent mutations and subsequent loss of protein expression (BAF250a) are a hallmark of EOC and are recognized as early molecular events in the malignant transformation of endometriosis [29,30]. This early involvement positions *ARID1a* downregulation as a highly relevant potential early biomarker for EOC development from ovarian endometriotic lesions. While *ARID1a* loss is strongly associated with EOC pathogenesis, its independent prognostic value remains debated, with some studies suggesting that its prognostic impact may be confounded by other factors, such as mismatch repair deficiency [31,32].

Moreover, our findings are particularly significant given that RIF1 overexpression has been consistently linked to aggressive disease, worse overall survival, and increased chemoresistance in epithelial ovarian cancer generally [33,34]. It should be emphasized that the protein expression analysis was limited to a small sample size. The known roles of RIF1 in DNA replication regulation and DNA repair pathways suggest that its dysregulation could contribute to genomic instability characteristic of cancer progression [35]. The observed expression pattern of RIF1 in our study, therefore, implicates it not only as a potential prognostic indicator but also as a marker reflecting the underlying mechanisms of tumor maintenance or resistance, warranting further investigation in the context of EOC progression.

Our observation that elevated *ARID1a* expression tends to be related to survival probability, although not statistically significant in stage IV EOC patients (HR = 0.44, *p* = 0.078), aligns with its established role as a tumor suppressor. *ARID1a* is typically linked to worse clinical outcomes [36,37,38,39]. Although our results reached only borderline significance, likely due to the limited sample size at late follow-up, the sustained separation of survival curves suggests that maintaining *ARID1a* expression may preserve genomic stability and suppress invasive progression [40]. Interestingly, while previous studies provided strong evidence that ARID1a loss is most prognostic in early-stage disease (FIGO I/II), our data support its potential relevance even in advanced stages [39,40].

In contrast, *RIF1* expression did not significantly correlate with overall survival. While high RIF1 expression has been linked to poorer survival in other EOC cohorts due to its role in promoting cell growth and telomerase activity, our findings suggest that its prognostic value may be overshadowed by other aggressive features in stage IV disease [34,41]. Another interesting finding of this study is the modest but statistically significant predictive performance of *RIF1* expression for platinum and taxane responsiveness (AUC = 0.568, *p* = 0.017). This is consistent with literature identifying *RIF1* as a critical mediator of DNA double-strand break repair choice [40]. *RIF1* promotes non-homologous end joining and inhibits the end-resection required for homologous recombination; consequently, its overexpression can confer resistance to DNA-damaging agents like cisplatin [41,42]. The trend toward lower *RIF1* expression in treatment responders observed in our study corroborates the hypothesis that reduced *RIF1* levels may sensitize cancer cells to chemotherapy-induced apoptosis by impairing repair mechanisms [43]. Notably, *ARID1a* showed no discriminatory capacity for therapeutic response (AUC = 0.504), highlighting that while *ARID1a* may influence long-term survival through tumor suppression, *RIF1* is a more relevant biomarker for immediate chemosensitivity in the clinical setting. The muTarget analysis revealed novel associations between specific mutational profiles and the expression levels of the genes *ARID1a* and *RIF1*. The significant reduction of *ARID1a* in *BRCA1*-mutant tumors (*p* = 2.82 × 10^−4^) is particularly noteworthy. While *ARID1a* and *BRCA1* mutations are often mutually exclusive with TP53 in certain subclasses of clear cell carcinoma, their co-occurrence or functional intersection in DNA repair pathways may represent a unique molecular phenotype [34,42,43].

Furthermore, the identification of *KIF17* as a potential positive regulator of *ARID1a* and of *LAMB3* as a regulator of *RIF1* expression provides new avenues for understanding the upstream oncogenic events that dictate the epigenetic landscape of EOC. *LAMB3* appears to function as an important oncogene in EOC, where its overexpression significantly promotes cellular proliferation, migration, and invasion via the WNT/β-catenin signaling pathway [44,45]. High *LAMB3* expression is clinically associated with poor prognosis and an increased metastatic propensity [44,45]. The biological connection between *LAMB3* mutations and the upregulation of the DNA repair factor, RIF1, is not direct or canonical, but several mechanistic links can plausibly connect extracellular matrix (ECM) dysfunction to genome instability and compensatory DNA damage responses [46]. ECM dysfunction can promote replication stress, and cells experiencing persistent replication stress often compensate by upregulating DNA repair machinery [47]. Thus, increased *RIF1* expression may reflect a secondary adaptive response to genome instability induced by disrupted epithelial-ECM homeostasis. The relationship is therefore likely to be indirect, but his interaction is clinically relevant. Consequently, the *LAMB3*-mediated potential association of *RIF1* represents a critical molecular intersection between extracellular matrix remodeling and genomic maintenance in EOC progression. Importantly, contemporary research has shown that the downregulation of *RIF1* enhances the sensitivity to platinum-based chemotherapy in epithelial ovarian cancer by regulating the nucleotide excision repair (NER) pathway [34]. The aforementioned study further revealed that *RIF1* knockdown potentiated cisplatin-induced apoptosis in epithelial ovarian cancer cells [34]. Consequently, *RIF1* represents a promising candidate as a novel molecular biomarker for predicting clinical response to platinum-based chemotherapy and for assessing the overall prognosis of patients diagnosed with EOC.

## 5. Conclusions and Limitations

In conclusion, our study offers a new perspective, emphasizing that EOC is a distinct clinical and molecular entity and identifies key molecular players. We demonstrated the expression patterns of *ARID1a*/BAF250a and *RIF1* in EOC and endometriosis, establishing their relevance in the context of tumor biology. Integrating *RIF1* as a predictive biomarker could enhance the precision of treatment selection in EOC, particularly for identifying patients likely to experience early relapse following standard platinum-based therapy. However, certain limitations, such as the small sample size of IHC samples and the retrospective character of this study, should be taken into consideration. The observational nature of the study and the lack of functional validation experiments are also limitations. Another limitation concerns the muTarget analysis of differential expression of ARID1a and RIF1 across the four most significant gene mutations, which was based on an ovarian cancer cohort with different subtypes, as it was not possible to restrict to EOC. Therefore, future studies could focus on the functional validation of the identified mutational drivers to determine if targeting these upstream regulators can restore *ARID1a* levels or suppress *RIF1*-mediated chemoresistance in EOC.

## Data Availability

Data will be made available on request.

## Abbreviations

The following abbreviations are used in this manuscript:

ARID1a	AT-rich interaction domain-containing protein 1a
BAF250a	BRG1/Brm-Associated Factor 250a
EOC	Endometrioid ovarian cancer
RIF1	Replication Timing Regulatory Factor 1
